# Supplementary material for: The nature of carotenoid S* state and its role in the nonphotochemical quenching of plants
Source: Nat Commun. 2024 Jan 29;15:847. doi: 10.1038/s41467-024-45090-9 (PMC11258248; doi:10.1038/s41467-024-45090-9)
Supplement: Supplementary file 1 — Supplementary Information [file 41467_2024_45090_MOESM1_ESM.pdf]

The nature of carotenoid S* state and its role in the nonphotochemical quenching of plants

Davide Accomasso*[1], Giacomo Londi[1], Lorenzo Cupellini[1], and Benedetta Mennucci*[1]

[1]Department of Chemistry and Industrial Chemistry, University of Pisa, 56124 Pisa, Italy
*Corresponding Author, e-mail: davide.accomasso@dcci.unipi.it, benedetta.mennucci@unipi.it

Supplementary Methods 1: Nonadiabatic excited state dynamics simulations

The starting conditions for the surface hopping (SH) simulations were sampled from the last 1.5 ps of 29 quantum mechanics/molecular mechanics (QM/MM) thermal equilibrations (see below). In the sampling procedure, the initial electronic state was selected according to the radiative transition probability from the ground state,[1] within an excitation energy window of 2.65 ± 0.15 eV, which includes most of the main band of the UV/vis absorption spectrum of lutein (Lut) in CP29, computed along the QM/MM equilibrations (Supplementary Fig. 9). In the SH simulations, a time step of 0.2 fs was employed in the integration of both the nuclear and electronic degrees of freedom. Quantum decoherence effects along the SH trajectories were approximately taken into account using the overlap decoherence correction scheme, with the following parameters: $\sigma = 1.0$ a.u. (Gaussian width) and $S_{min} = 5 \times 10^{-3}$ (minimum overlap). The six lowest singlet electronic states were taken into account in the nonadiabatic SH dynamics. For each simulation time, the population of each electronic state i was computed as the fraction of SH trajectories running on the i-th potential energy surface. The single-point calculations to estimate the $S_1 \rightarrow S_n$ excited state absorption of Lut were performed at the same semiempirical QM/MM level of theory used in the SH simulations, including up to 10 electronic states. Energy gap and transition dipole moments were computed for the 10 lowest singlet electronic states of Lut and the absorption spectrum was then obtained by summing the individual $S_1 \rightarrow S_n$ contributions (n = 5–9).

Supplementary Methods 2: Thermal equilibrations

Thermal equilibrations in the ground state (S_0) were performed to produce an ensemble of nuclear coordinates and velocities of Lut in CP29. In these simulations, we used the same QM/MM electrostatic embedding scheme as employed in the SH simulations. We performed 29 QM/MM

thermal equilibrations: 19 for set A and 10 for set B. Each QM/MM equilibration was propagated for 2 ps using the Bussi-Parrinello thermostat[2], with a temperature of 300 K, a time step of 0.5 fs, and a relaxation time constant of 1 fs. Additional data obtained from the QM/MM thermal equilibrations are provided in Supplementary Fig. 14-15.

Supplementary Note 1: Fitting of the S_1 state population

In order to extract the lifetime for the S_1 state, the S_1 population obtained in the SH simulations for Lut in CP29 was fitted using the following rate model:

$$\mathrm{S_2, S_3} \xrightarrow{\tau_2} \mathrm{S_1} \xrightarrow{\tau_1,\ \tau_1'} \mathrm{S_0} \tag{1}$$

where τ_1, τ_1', and τ_2 are time constants. In particular, the decay of the initially populated S_2 and S_3 states was modelled by a single exponential function:

$$S_{2,3}(t) = P_{2,3}(0)\ e^{-t/\tau_2}. \tag{2}$$

where $P_{2,3}(0)$ is the sum of initial (t = 0) populations of S_2 and S_3. A bi-exponential decay of the intermediate state S_1 was assumed and the S_1 population was fitted using the following function:

$$\begin{aligned} S_1(t) = {} & w\Big[P_1(0)\ e^{-t/\tau_1} + P_{2,3}(0)\ \frac{\tau_1}{\tau_1-\tau_2}\ \big(e^{-t/\tau_1} - e^{-t/\tau_2}\big)\Big] + \\ & + (1-w)\Big[P_1(0)\ e^{-t/\tau_1'} + P_{2,3}(0)\ \frac{\tau_1'}{\tau_1'-\tau_2}\ \big(e^{-t/\tau_1'} - e^{-t/\tau_2}\big)\Big] \end{aligned} \tag{3}$$

in which $P_1(0)$ is the initial population of S_1, and w and 1-w are the weights of the two exponential components ($0 \leq w \leq 1$), with time constants for S_1 decay τ_1 and τ_1', respectively. In the fitting procedure, τ_1, τ_1', τ_2 and w were determined by fitting the S_1 population using function $S_1(t)$ (Supplementary Equation 3). Then, the average decay time for S_1 (τ_1^{avg}) was computed as follows: $\tau_1^{avg} = w\ \tau_1 + (1-w)\ \tau_1'$.

Supplementary Note 2: Distortion of the conjugated chain

To quantify the distortion of the π-conjugated chain of Lut, the following formula was used:

$$D = \frac{1}{2N_{dih}} \sum_{i=1}^{N_{dih}} \big[1 + cos(2\phi_i - \pi)\big] \tag{4}$$

where N_{dih} is the total number of dihedral angles considered and ϕ_i are the dihedral angles values. D ranges from 0 for an all-planar arrangement (whether cis or trans) to 1 for the case where all the dihedrals are equal to 90°.

Supplementary Figures

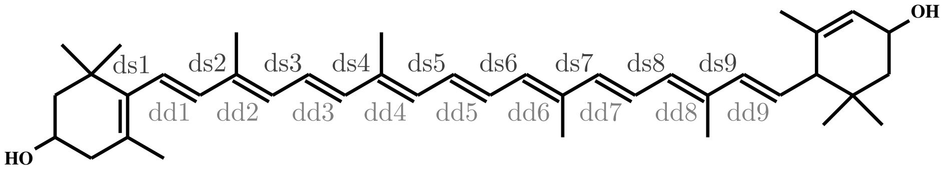

Supplementary Fig. 1: **Molecular structure of lutein**. Labels refer to the dihedral angles of the π-conjugated C−C and C=C bonds (ds1-9 and dd1-9, respectively).

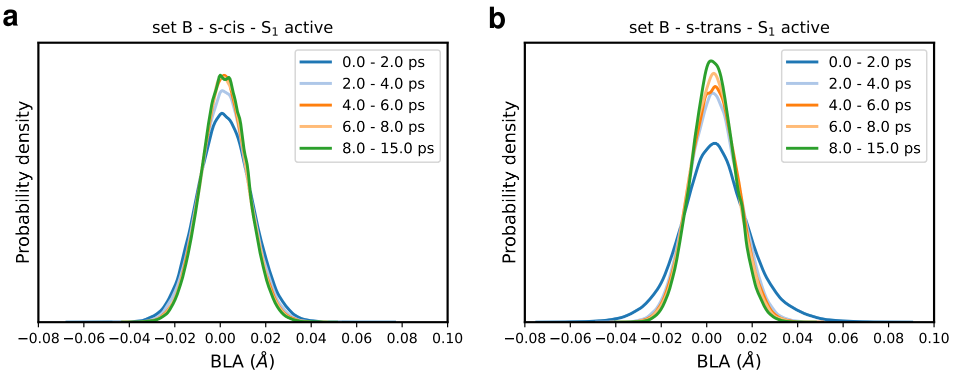

Supplementary Fig. 2: **Distributions of the bond-length alternation (BLA, in Å)**. Values refer to the lutein (Lut) S_1 state for different time intervals of the surface hopping simulations for set B: **a** Lut s-cis; **b** Lut s-trans.

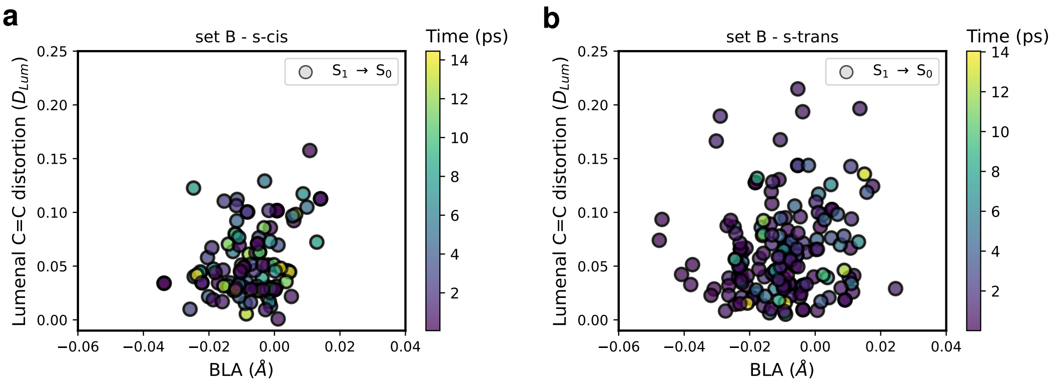

Supplementary Fig. 3: **Relationship between C=C distortion and BLA.** Distortion around the C=C bonds of the lumenal side (D_{Lum}, Supplementary Equation (4) for dihedral angles dd1-dd4) of lutein (Lut) versus the bond-length alternation (BLA, in Å) at the $S_1 \rightarrow S_0$ hops of the surface hopping simulations for set B: **a** Lut s-cis; **b** Lut s-trans. Dots are colored according to the $S_1 \rightarrow S_0$ hopping time (in ps).

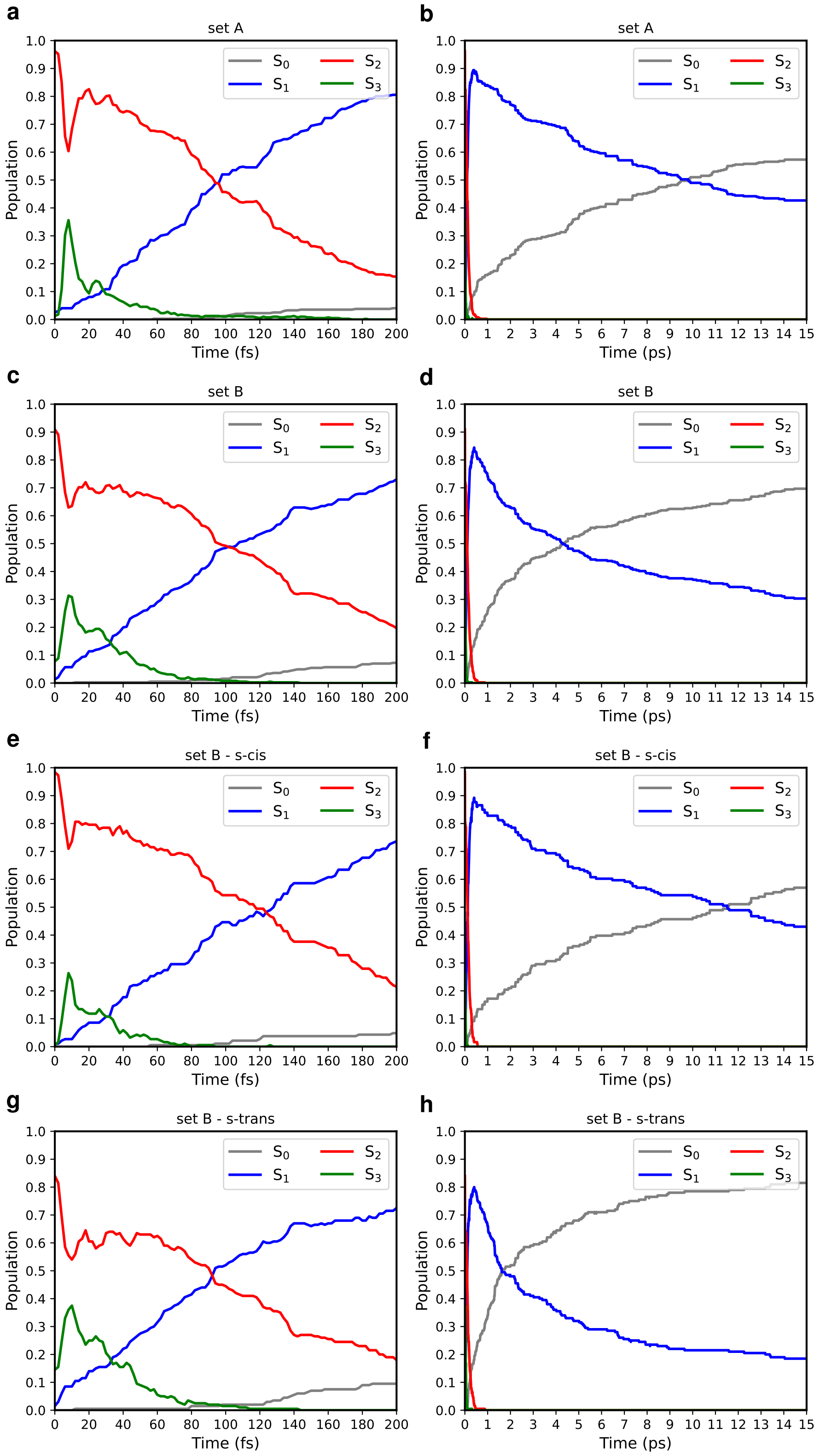

Supplementary Fig. 4: **State populations as a function of time (in fs)**. Populations were obtained in the surface hopping simulations for lutein in CP29: **a, b** set A; **c, d** set B; **e, f** set B - s-cis; **g, h** set B - s-trans. Results were obtained by averaging over all trajectories and time intervals of 2 fs.

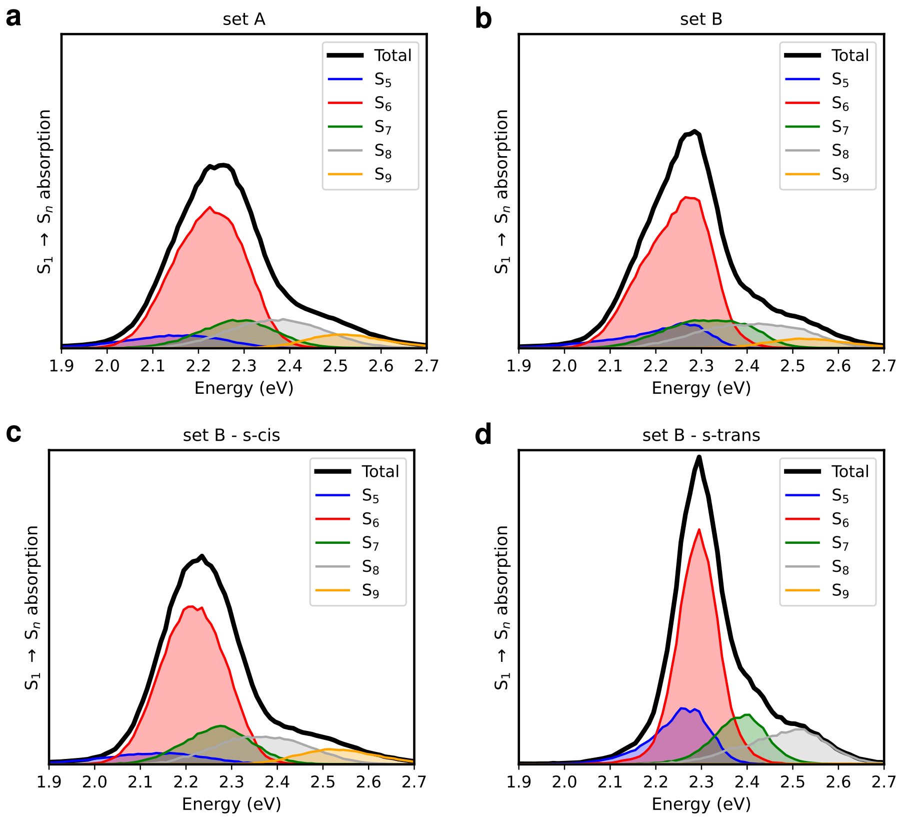

Supplementary Fig. 5: **The $S_1 \rightarrow S_n$ excited state absorption spectra of lutein**: **a** set A; **b** set B; **c** set B - s-cis; **d** set B - s-trans. The contributions from the different S_n states (S_5-S_9) were averaged over all the surface hopping trajectories and the whole simulation time of 15 ps.

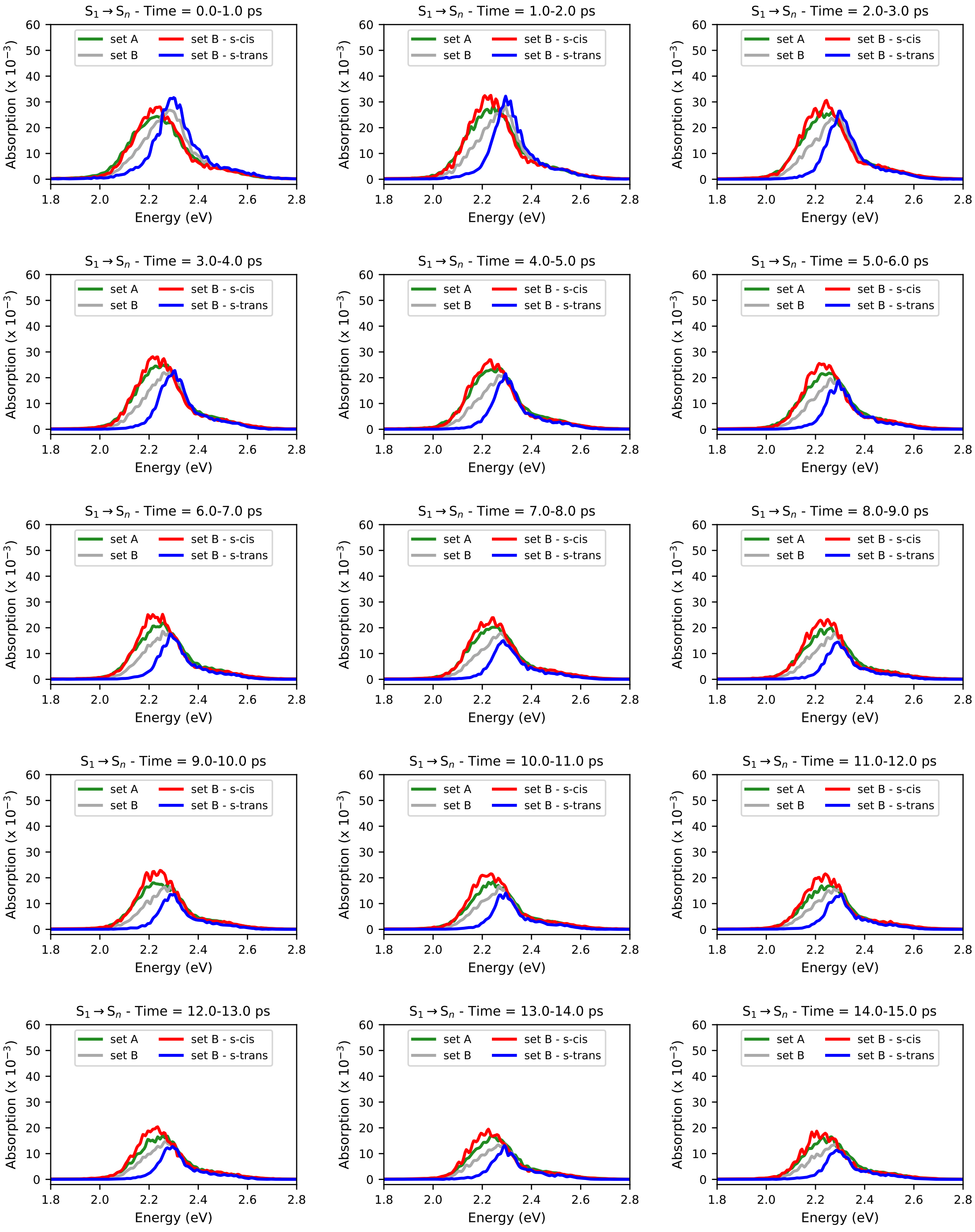

Supplementary Fig. 6: **The S$_1$ $\rightarrow$ S$_n$ absorption spectra**. Spectra were averaged over different time intervals obtained in the surface hopping simulations for lutein in CP29.

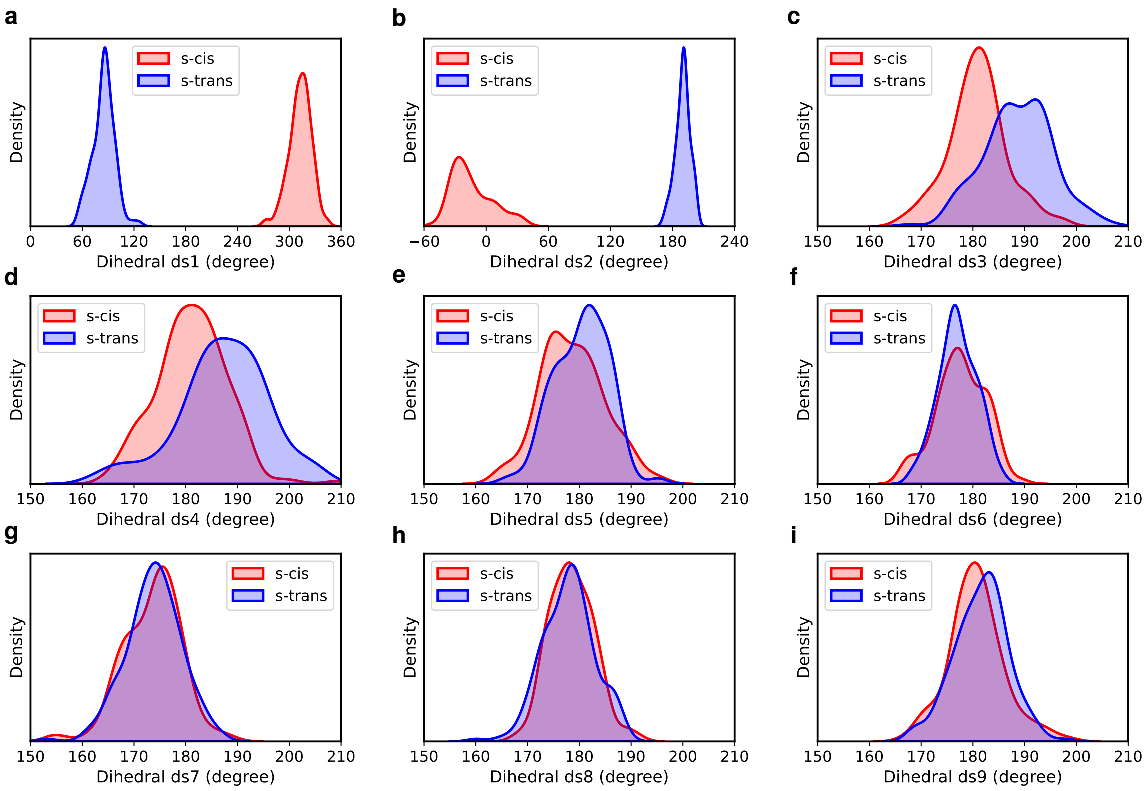

Supplementary Fig. 7: **Distributions of the dihedral angles around the C-C bonds**: **a-i** dihedral ds1-ds9 of lutein s-trans (in blue) and s-cis (in red) conformers in the starting geometries of the surface hopping trajectories for set B.

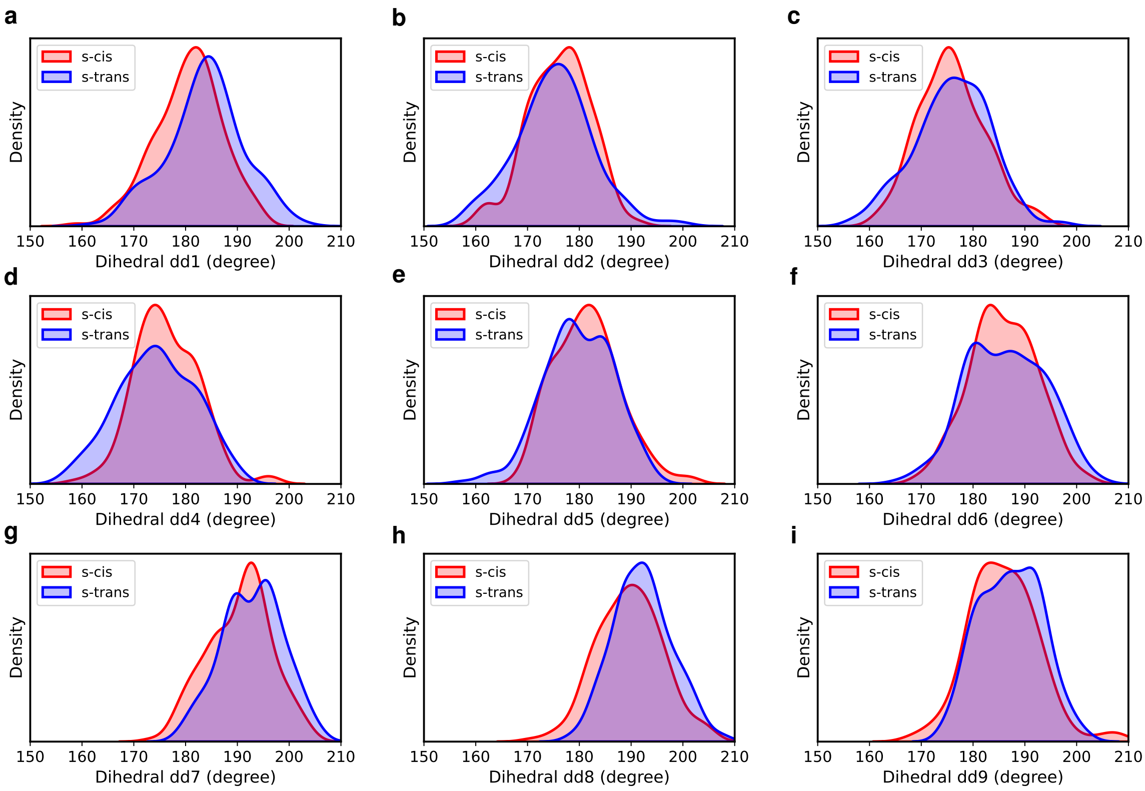

Supplementary Fig. 8: **Distributions of the dihedral angles around the C=C bonds**: **a-i** dihedral dd1-dd9 of lutein s-trans (in blue) and s-cis (in red) conformers in the starting geometries of the surface hopping trajectories for set B.

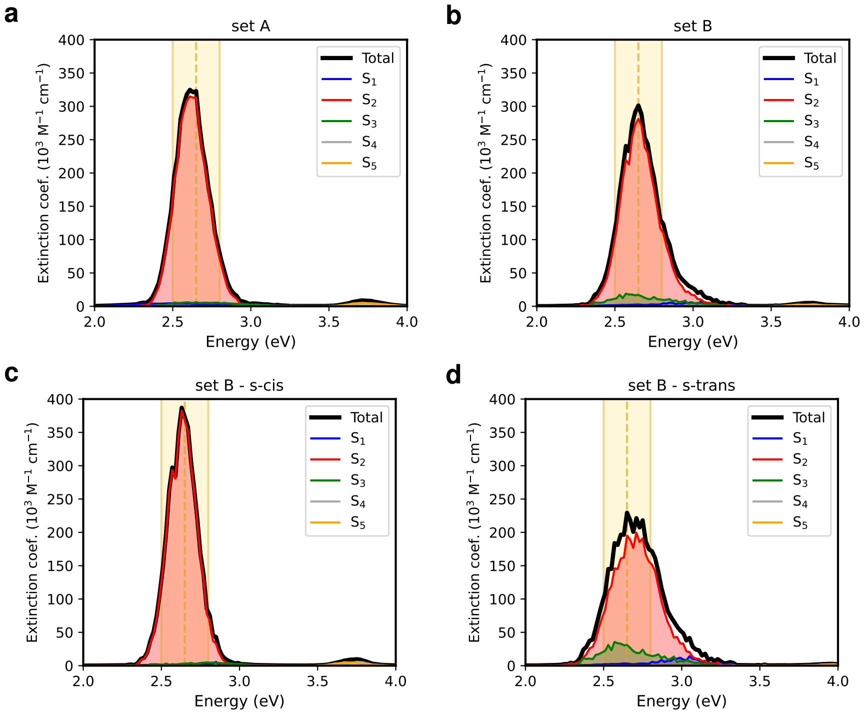

Supplementary Fig. 9: **The absorption spectra of lutein in CP29 computed along the QM/MM thermal equilibrations**: **a** set A; **b** set B; **c** set B - s-cis; **d** set B - s-trans (time interval from 0.5 ps to 2.0 ps). The contributions of the five lowest singlet excited states (S_1-S_5) are shown, together with the energy window (in gold) employed in the sampling of initial conditions for the surface hopping simulations.

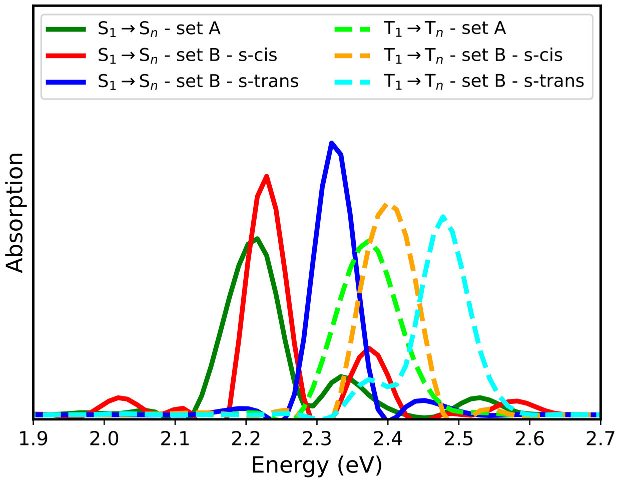

Supplementary Fig. 10: **The $S_1 \rightarrow S_n$ and $T_1 \rightarrow T_n$ excited state absorption spectra of lutein (Lut)**. Spectra were computed using 19 and 10 structures for set A and set B, respectively, optimized at the S_1 and T_1 states of Lut at the semiempirical QM/MM level.

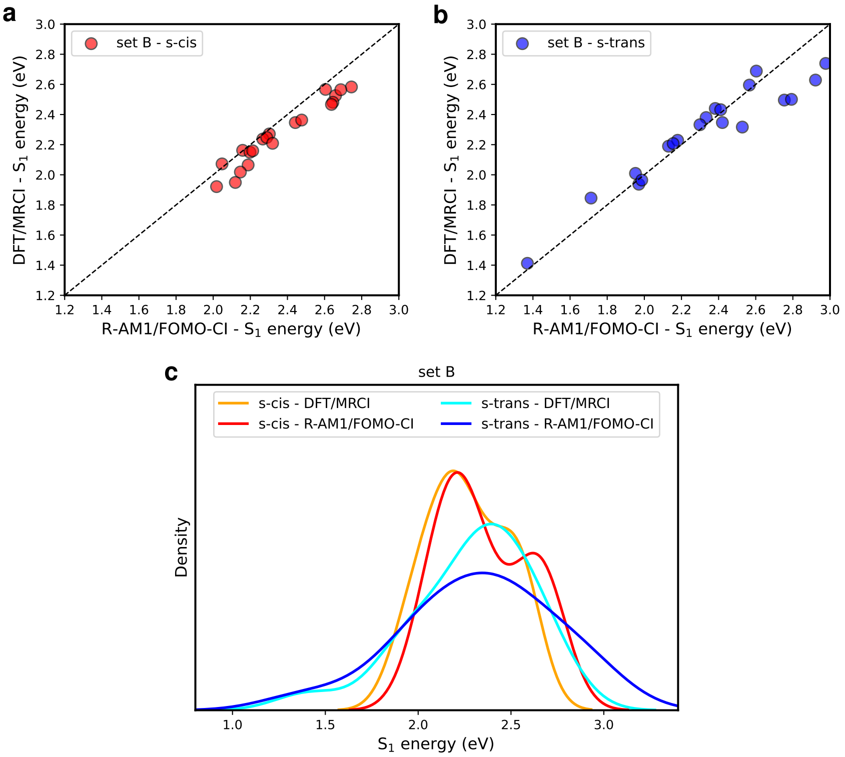

Supplementary Fig. 11: **Comparison of the vertical S_1 excitation energies for lutein (Lut).** Values were computed at the semiempirical R-AM1/FOMO-CI versus DFT/MRCI levels for structures of set B selected from the QM/MM thermal equilibrations: **a** Lut s-cis; **b** Lut s-trans. Mean deviations of the R-AM1/FOMO-CI S_1 excitation energies from the DFT/MRCI energies are 0.04 eV for Lut s-trans and 0.09 eV for Lut s-cis, while mean absolute deviations are 0.10 eV for Lut s-trans and 0.09 eV for Lut s-cis. **c** Distributions of the vertical S_1 excitation energies computed at the two different levels of theory for Lut s-trans and s-cis of set B.

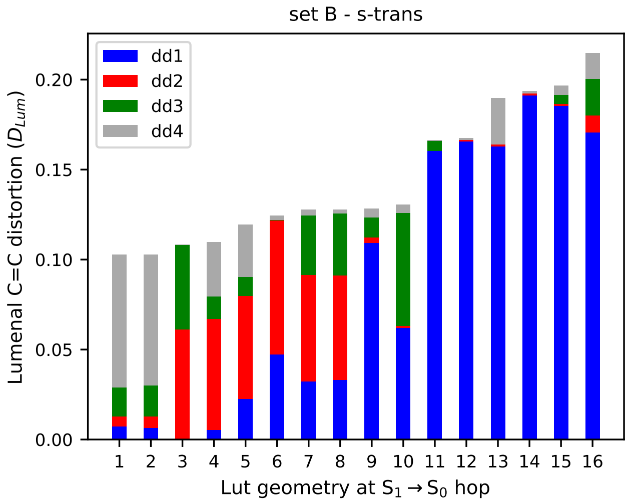

Supplementary Fig. 12: **Contributions of the individual dihedral angles (dd1-dd4) to D_{Lum} of lutein s-trans in set B**. Data refer to the $S_1 \rightarrow S_0$ hopping geometries with the largest distortion (*i.e.*, only the geometries in the red inset of Fig. 5d in the main text were considered).

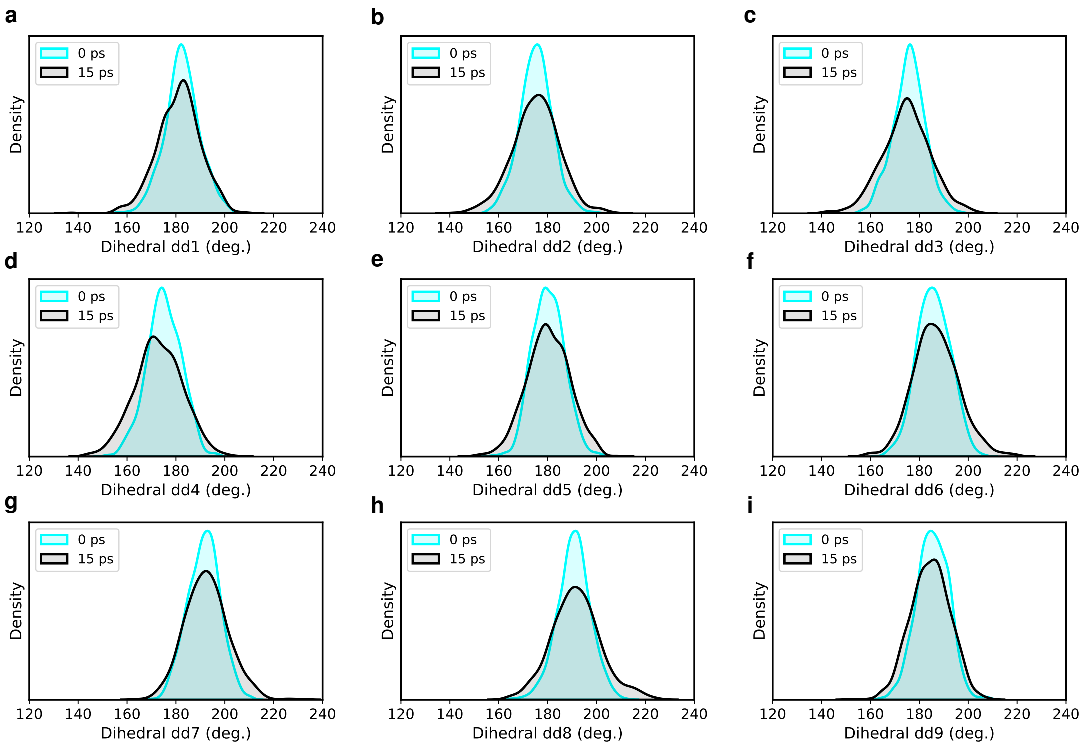

Supplementary Fig. 13: **Distributions of the dihedral angles around the C=C bonds**: **a-i** dihedral dd1-dd9 of lutein in the starting (0 ps, in cyan) and final (15 ps, in black) geometries of the surface hopping trajectories for both sets A and B.

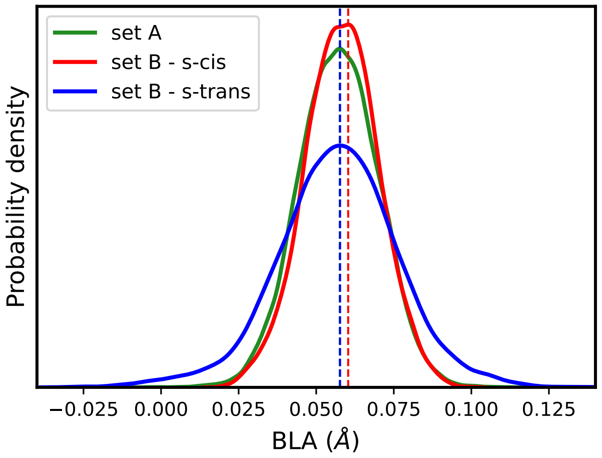

Supplementary Fig. 14: **Distributions of the bond-length alternation (BLA, in Å)**. Values were obtained in the QM/MM thermal equilibrations of lutein in CP29 for set A (in green), set B s-cis (in red), and set B s-trans (in blue) (time interval from 0.5 ps to 2.0 ps).

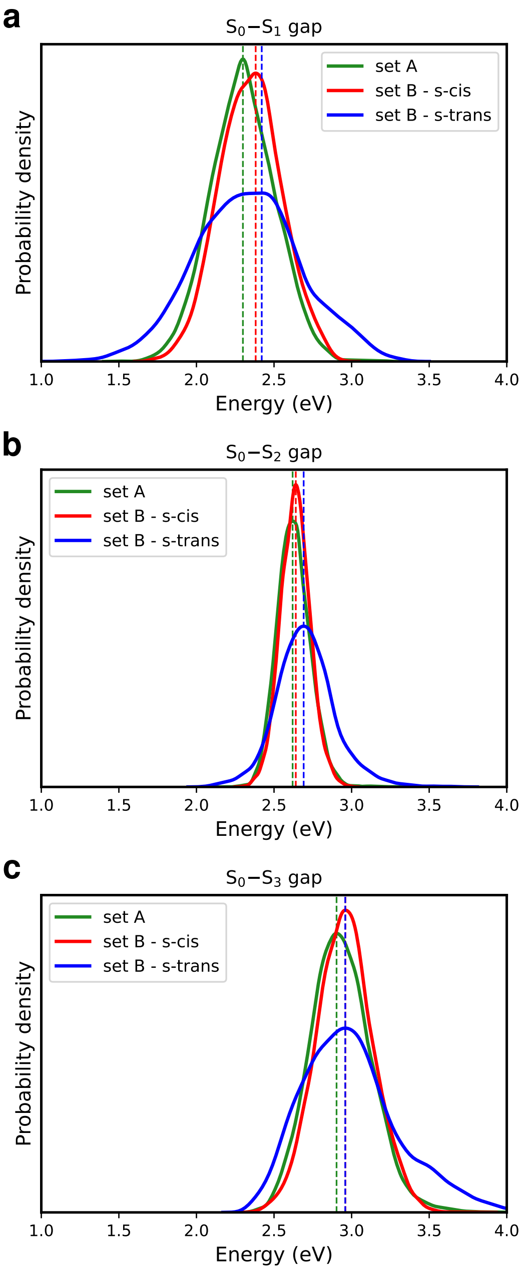

Supplementary Fig. 15: **Distributions of vertical excitation energies (in eV) from the ground state S_0 to**: **a** S_1; **b** S_2; **c** S_3 obtained in the QM/MM thermal equilibrations of lutein in CP29 for set A (in green), set B s-cis (in red), and set B s-trans (in blue) (time interval from 0.5 ps to 2.0 ps).

Supplementary Tables

Supplementary Table 1: Total number of surface hopping trajectories and their partition according to the starting electronic state of lutein in CP29.

Set	# Traj.	Starting state		
		S_1	S_2	S_3
A	396	10	381	5
B	386	5	351	30
B - s-cis	186	2	183	1
B - s-trans	200	3	168	29

Supplementary Table 2: Vertical S_1 excitation energy of lutein (Lut) computed along the QM/MM thermal equilibrations in CP29 (time interval from 0.5 ps to 2.0 ps). For each set, the mode (*i.e.*, the most frequent value), the mean, and the standard deviation of Lut S_1 energy are reported.

Set	S_1 energy (eV)		
	Mode	Mean	Std. Dev.
A	2.296	2.302	0.210
B - s-cis	2.381	2.347	0.200
B - s-trans	2.407	2.331	0.342

Supplementary Table 3: Average number of moving and frozen MM atoms in the QM/MM simulations of lutein in CP29 for sets A, B, B - s-cis, and B - s-trans.

Set	MM atoms		
	Total	Moving	Frozen
A	19200	10176 (53.0%)	9024 (47.0%)
B	19408	10134 (52.2%)	9274 (47.8%)
B-cis	19388	10118 (52.2%)	9270 (47.8%)
B-trans	19428	10150 (52.2%)	9278 (47.8%)

Supplementary Table 4: Main electronic configurations (h for HOMO and l for LUMO) of S_1 of lutein s-trans and s-cis in set B, computed along the surface hopping trajectories running on the S_1 state. For each configuration, the corresponding average weight in the S_1 electronic wave function and the average oscillator strength for the $S_1 \rightarrow S_0$ transition is reported.

Configuration	Weight		Oscillator strength	
	s-cis	s-trans	s-cis	s-trans
$h, h \rightarrow l, l$	0.322	0.313	0.003	0.008
$h \rightarrow l + 1$	0.255	0.253		
$h - 1 \rightarrow l$	0.223	0.228		

Supplementary References

[1] Persico, M. & Granucci, G. An overview of nonadiabatic dynamics simulations methods, with focus on the direct approach versus the fitting of potential energy surfaces. *Theor. Chem. Acc.* **133**, 1526/1–1526/28 (2014).

[2] Bussi, G., Donadio, D. & Parrinello, M. Canonical sampling through velocity rescaling. *J. Chem. Phys.* **126**, 014101 (2007).
